# First Study of Improved Nutritional Properties and Anti-Oxidant Activity in Novel Sesame Mutant Lines as Compared to Their Wild-Types

**DOI:** 10.3390/plants11091099

**Published:** 2022-04-19

**Authors:** Mohamed Kouighat, Abdelghani Nabloussi, Atman Adiba, Mohamed El Fechtali, Hafida Hanine

**Affiliations:** 1 Research Unit of Plant Breeding and Plant Genetic Resources Conservation, Regional Agricultural Research Center of Meknes, National Institute of Agricultural Research, Avenue Ennasr, P.O. Box 415, Rabat 10090, Morocco; mohammed.kouighat@usms.ac.ma (M.K.); adiba.atman@gmail.com (A.A.); mohamed.elfechtali@inra.ma (M.E.F.); 2 Laboratory of Bioprocess and Biointerfaces, Department of Biology, Faculty of Sciences and Technics, University Moulay Slimane, P.O. Box 523, Beni-Mellal 23000, Morocco

**Keywords:** sesame mutant, nutritional quality, antioxidant capacity, medicinal plant

## Abstract

Sesame seed represents a reservoir of nutritional components with many medicinal properties. With the current trend to increase both seed yield and nutritional quality, the cultivation of new high-quality sesame varieties is a necessity to improve human health and promote the economic efficiency of this crop. However, research efforts for the development of cultivars of high nutritional quality are too scarce. In this study, we evaluated the nutritional value and antioxidant activity of seeds of selected M_3_ sesame mutants, in comparison with their two wild-type cultivars. The measurements included ash, proteins, crude fibers, sugars, total phenolic content (TPC), total flavonoid content (TFC), total anthocyanin content (TAC), lignans and free radical scavenging activity (FRSA). The results show higher FRSA, TPC, TAC and lignans in the mutant “US2-6”, compared to the wild type “US06”. Besides this, seeds of the mutant “US1-DL” are rich in ash and sugars, while high protein and fiber contents were found in the mutants “ML2-5” and “US2-7”, respectively. This work highlights the possibility of improving the nutritional value of sesame germplasm through mutagenesis. The valuable germplasm obtained will be used in the sesame breeding program to develop cultivars with high nutritional quality and antioxidant activity, which could contribute to the prevention of diseases related to free radicals and nutritional deficiencies.

## 1. Introduction

Medicinal plants are important sources of medicines and aromas. Overall, thousands of native plants are used to cure and manage many diseases [1]. Among them, sesame (*Sesamum indicum* L.) is an annual plant that belongs to the Pedaliaceae family [1,2], and is also considered as an oilseed crop known by several names: beniseed, sesame, sesamum, gingelly, sim-sim and til [3].

Sesame is particularly cultivated for its high-quality nutritional seeds [4]. The nutritional importance of its seeds is due to the presence of high contents of oil (30–62%), proteins (18–20%), carbohydrates (13.4–25%), digestible fibers (>9.8%), calcium (1.92%), magnesium (0.14%), potassium (0.67%) and iron (0.03%) [5]. Sesame seeds are also a good source of antioxidants such as carotenoids, flavonoids, lignans, polyphenols and sterols [6]. These bioactive compounds facilitate plant survival and promote human health [7,8]. Lignans play an important role in the metabolism of lipid and glucose, the prevention of hypertension, and the reduction of bad cholesterol, and the have anti-cancer, anti-inflammatory, neuroprotective, bactericidal and insecticidal, and antifungal activities [9]. Flavonoids are a large group of phytonutrients, widely distributed in various plant sources, that cannot be synthesized by humans [10,11]. Most flavonoids are potent antioxidants with anticancer, anti-aging, anti-inflammatory, anti-hepatotoxic, antitumor, antimicrobial, antithrombotic and antiviral properties [11,12]. Polyphenols represent the main and most predominant group of primary antioxidants [13,14] with anticarcinogenic properties [15] and anti-inflammatory activity [16].

With the increase in health awareness, people are more concerned about food nutritional quality. To meet dietary requirements, sesame seed quality should be further improved. The cultivation of new high-quality sesame varieties is of great importance for improving health and nutrition, promoting market consumption, boosting economic efficiency, and accelerating the growth of the sesame industry [17]. Attempts to improve the seed quality of sesame by mutagenesis have been reported. However, to our knowledge, they are scarce and limited to fatty acids composition [18] and lignan profile [19,20].

In Morocco, the genetic diversity of sesame is very limited [21,22], and therefore, mutagenesis breeding using EMS (ethyl methane sulfonate) was designed and implemented to enlarge the existing variability [23]. As a result, some promising mutants have been identified and isolated. They exhibit a high level of tolerance to drought during the seed germination and early seedling growth stage [24]. However, no information is available on the nutritional and medicinal quality of these genetic materials. The existing Moroccan cultivars present good quality, both in seeds [25,26] and in oil [27], but this is not enough to meet the expectations related to health and nutrition concerns.

The present study is an assessment of 13 promising mutant lines, along with their wild-type parents, based on the most important nutritional quality traits. Besides the known traditional parameters mentioned above, the present work approached for the first time the anthocyanins content. Anthocyanins are natural pigments with high antioxidant, anti-inflammatory and neuroprotective properties, which maintain cholesterol and blood sugar at an appropriate level and prevent cancers [6,28,29,30]. Therefore, the objective of this study was to evaluate the 13 mutant lines for various chemical and biochemical markers, against their parental cultivars, and to identify and select the most interesting genetic materials with high nutritional quality and antioxidant activity.

## 2. Results

### 2.1. Variability among the Investigated Genotypes

Highly significant differences (*p* < 0.01) were observed among the lines in terms of lignans, TFC, TPC, TAC and DPPH reduction capacity, while significant differences (*p* < 0.05) were shown for carbohydrate, crude fiber and protein contents (Table 1). This indicates there is a genetic diversity among the obtained mutants and their parents. However, no significant difference was found for ash content.

The results of the ANOVA–planned contrast analysis showed significant differences between the parents and their respective mutants (The parent ML13 vs. ML-mutants and the parent US06 vs. US-mutants), and between the ML-genotypes group and the US-genotypes group (ML type vs. US type) (Table 1). In fact, the ML-mutants group differed significantly from to the wild-type parent ML13 for all the traits studied. Additionally, significant differences were observed between the parent US06 and the US-mutants group for all the investigated parameters. Similarly, there were significant differences between ML type and US type for all the parameters. The ash content was excluded from this contrast analysis as ANOVA results did not show any genotype effect on this parameter.

### 2.2. Ash Content

The overall ash content was 4.42, with a variation from 4.15 to 5.1%. However, this variation was not statistically significant, indicating all the mutant lines and their wild types were comparable for this parameter.

### 2.3. Carbohydrate Content

**Figure 1:**
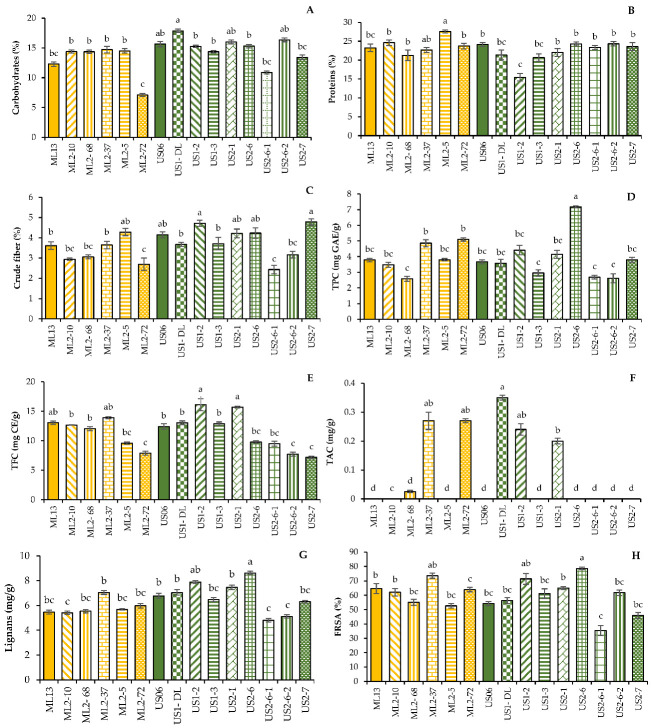
Differences among 13 M_3_ sesame mutants and their wild-parents (“US06” and “ML13”) for some nutritional parameters and antioxidant activity: carbohydrate content (**A**), protein content (**B**), crude fiber content (**C**), TPC or total polyphenol content (**D**), TFC or total flavonoid content (**E**), TAC or total anthocyanin content (**F**), total lignans content (**G**)**,** and percentage of FRSA or free radical scavenging activity (**H**). Values with the same alphabetical superscripts are not significantly different according to the DMRT test (*p* > 0.05).

The carbohydrates content in the investigated seeds ranged from 7.12 to 17.87%, with an average value of 14.16% (Figure 1A). The white seeds of the mutant “US1-DL” contained the highest amount of carbohydrate, 17.87%, which is 2.23% higher than the wild type US06 (15.64%). However, the brown seeds of the mutant “ML2-72” showed the lowest carbohydrate content (7.12%), compared to seeds of the wild parent “ML13”, with 12.28%. Overall, the carbohydrate content of dark seeds (US2-6, ML2-72 and ML2-37) was lower than that of white seeds (US1-DL, US2-6-2 and US2-1) (Figure 1A).

### 2.4. Protein Content

The protein content differs significantly (*p* < 0.05) in the 15 genotypes, ranging from 15.47 to 27.58% (Figure 1B). The highest protein content (27.58%) was registered in the mutant “ML2-5”, which was 4.33% higher than that of the wild type “ML13” (23.25%). Additionally, the “ML2-10” mutant contained 1.36% more protein than the “ML13” parent having an average content of 24.61%. On the other hand, there was a drastic reduction in the mutant “US1-2”, exhibiting less than 15.50%, which is much lower than the wild-type “US06” containing more than 24%. For the other mutant lines, the protein contents were similar to those of the wild-type parents.

### 2.5. Crude Fiber Content

Crude fiber content varied significantly (*p* < 0.05) from 2.43 to 4.78% in the mutant lines “US2-6-1” and “US2-7”, respectively (Figure 1C). The wild-type parent “US06” contained 4.15% of this crude fiber. The other wild-type parent “ML13” contained a percentage of 3.61%, while the best mutant line, “ML2-5”, showed an increased content of 4.28%.

### 2.6. Total Phenol Content

The mean values of total phenol content (TPC) in the different genotypes are presented in Figure 1D. The TPC in sesame seeds tested ranged from 2.58 to 7.17 mg GAE/g. The highest TPC value was observed in the black seeds of the mutant “US2-6” (7.17 mg GAE/g), almost double that found in the white seeds of the wild parent “US06” (3.66 mg GAE/g). Similarly, compared to the wild-type parent “ML13” (3.78 mg GAE/g), the brown seeds of the mutants “ML2-72” and “ML2-37” contain more phenols, with a respective content of 5.1 and 4.86 mg GAE/g. The lowest value (2.58 mg GAE/g) was obtained in the seeds of the mutant “ML2-68”.

### 2.7. Total Flavonoid Content

Total flavonoid content (TFC) was significantly affected by genotype (Table 2), ranging from 7.18 to 16.09 mg CE/g in the mutants “US2-7” and “US1-2”, respectively (Figure 1E). The wild-type parent US06 contains only 12.41 mg CE/g, revealing that TFC was enhanced by 3.68 mg CE/g in seeds of the mutant line “US1-2”. The lowest TFC (7.18 mg CE/g) was found in the mutant US2-7, which is 5.23 mg CE/g less than that of the parent US06. Interestingly, only seeds of the mutants “US1-2” and “US2-1” were more flavonoid-rich than those of the parent, with average contents of 16.09 and 15.59 mg CE/g, respectively. For the rest of the mutant lines, the TFC was either maintained or reduced, compared to the wild-type parents.

### 2.8. Total Anthocyanin Content

As shown in Figure 1F, anthocyanin exists only in pigmented sesame seeds. The highest TAC value, 0.35 mg/g, was found in the black seeds of the mutant “US2-6”, followed by 0.27 mg/g in the gray seeds of the mutant “ML2-68”, as well as in the brown seeds of the mutant “ML2-37”. Other mutants, namely “ML2-5”, “ML2-37” and “ML2-10” showed an average content of 0.24, 0.2 and 0.025 mg/g, respectively. However, no anthocyanin was detected in white and beige seeds from the rest of the studied genotypes, including both wild-type parents.

### 2.9. Total Lignan Content

The total lignan content in seed extracts of the 15 genotypes is presented in the Figure 1G. Lignan content was detected in extracts of all genotypes, with a significant variation from 4.81 to 8.61 mg/g in the mutants “US2-6-1” and “US2-6”, respectively, while the wild-type parent “US06” contains 6.78 mg/g of total lignan. Similarly, compared to the other wild-type parent “ML13” containing 5.46 mg/g of total lignan, the mutant “ML2-37” showed a higher concentration, with an average value of 7.06 mg/g.

### 2.10. Free Radical Scavenging Activity

The results of free radical scavenging activity (FRSA) were expressed as percentage of inhibition and are summarized in the Figure 1H. A significant variation was observed among the genotypes studied for this parameter. The percentage of antioxidant activity ranged from 35.44 to 78.62%. It was the mutant “US2-6” that showed the highest percentage of inhibition (78.62%), followed by “ML2-37” (73.47%) and “US1-2” (71.35%), against the wild-type parents “US06” and “ML13”, having an average percentage of 54.18 and 64.61%, respectively.

### 2.11. Correlation between Biochemical Attributes

Table 2 shows the Pearson’s correlation coefficients between the different traits studied. FRSA had a positive, strong and highly significant correlation with TPC (r = 0.850, *p* < 0.01), TAC (r = 0.712, *p* < 0.01) and TFC (r = 0.656, *p* < 0.01). Addtionally, but to a lesser degree, TPC was positively and significantly associated with TAC (r = 0.479, *p* < 0.05), TFC (r = 0.398, *p* < 0.05) and total lignan content (r = 0.398, *p* < 0.05). These correlations were expected since the increase in TPC, TAC and TFC is generally associated with elevated antioxidant activity. These results show that the antioxidant capacity of sesame seeds is generally due to the presence of high levels of TPC, TAC, and TFC. Besides this, a high polyphenols concentration is found to be associated with high lignan content.

## 3. Discussion

In the present study, the significant differences observed between each parent and its mutants indicate the effectiveness of EMS mutagenesis breeding in expanding and improving the genetic variability in the investigated biochemical parameters. Additionally, the significant variation found between the ML type and the US type demonstrates the usefulness of mutagenizing seeds of both cultivars ML13 and US06 for enhancing and developing various novel sesame germplasms with improved nutritional properties. Overall, a genetic gain was found in most mutant lines, compared to the wild-type parents, for all studied traits except ash content. For carbohydrates content, the observed variation range (7.12–17.87%) was higher than that reported by El Khier et al. [31] (1.05–2.88%), Yaseen et al. [32] (7.7–12.6%) and Zebib et al. [33] (8.3–11.69%). The highest content was recorded in the mutant “US1-DL” (17.87%), indicating a significant superiority of 2.23% over the wild-type parent “US06” (15.64%). This mutant is also characterized by white seeds, thus confirming that the carbohydrates contained in the white sesame seeds are higher than dark seeds [34,35]. Therefore, seeds of this mutant line could be used as a good food source for daily carbohydrate intake.

Regarding the protein content, there was also an improvement as a result of mutagenesis breeding. Protein content in the seeds of the mutants “ML2-5” (27.58%) and “ML2-10” (24.61%) are raised by 4.33 and 1.36%, respectively, compared to the parent “ML13” (23.25%). This genetic gain allowed for reaching protein contents that are higher than those found by Gharby et al. [26] (22%), Hassan [36] (18%), Moazzami and Kamal-Aldin [37] and Ünal and Yalçın [38] (21%). On the other hand, our results are lower than those reported by Nzikou et al. [39] and Ogbonna and Ukaan [40] (27.90–34.4%). Nevertheless, in addition to their improved nutritional quality, our mutant lines (ML2-5 and ML2-10) are also characterized by their tolerance to severe drought during germination and early seedling growth [24]. Thus, these two genotypes accumulating beneficial traits of both quality and drought tolerance may be relevant and interesting germplasms for a sesame breeding program aimed at improving the agronomic, nutritional, and medicinal properties of this crop.

For the crude fiber content, the mutant lines “US2-7” (4.78%) and “US1-2” (4.71%) showed the highest values, which were 0.63 and 0.55% more than that of their parent “US06” (4.15%). Although this improved content is higher than that reported by El Khier et al. [31] (3.3–4.67%), it remains much lower than what was found by Obiajunwa et al. [41] (3.2–10.0%) and Abbas et al. [42] (3.62–8.77%). Nonetheless, the mutants “US2-7” and “US1-2” are also interesting given their richness in TFC, as will be shown below, suggesting that their seeds could be a food of choice for improving human health. Therefore, these lines may be considered as relevant germplasm for breeding and developing cultivars with more than one interesting trait from a nutritional and medicinal point of view.

The total phenolic content (TPC) varied significantly from 2.58 to 7.17 mg GAE/g among the lines studied. This variation is broader than the range found by Khan et al. [43] (1.64–3.32 mg EAG/g), Kurt et al. [44] (1.99–6.81 mg EAG/g) and Rizki et al. [25] (3.75–3.92 mg EAG/g). On the other hand, our values are more or less similar to those of Zhou et al. [45] (4.54–7.32 mg GAE/g) and Lin et al. [30] (3.7–7.8 mg GAE/g). One could notice that TPC in the mutant “US2-6” (7.17 mg GAE/g), characterized by black seeds, was almost double that of the wild-type parent “US06” (3.66 mg GAE/g), with white seeds. Likewise, compared to the wild-type parent “ML13” (yellow seeds), the brown seeds of mutants “ML2-72” and “ML2-37” contain more total polyphenol, with plus 1.3 and 0.24 mg GAE/g, respectively. These results corroborate previous findings that demonstrated that polyphenols are abundant in dark seed coats [30,46,47,48,49]. It is also important to note that, for lignan content, the mutant lines “US2-6” (8.61 mg/g) and “ML2-37” (7.06 mg/g) showed 1.83 and 1.6 mg/g more than the respective parents, “US06” (6.78 mg/g) and “ML13” (5.46 mg/g). In previous works on sesame, it was reported that the lignan content varied from 4.05 to 11.78 mg/g [50] and 2.52 to 12.76 mg/g [51]. The richness in both TPC and lignan is significantly correlated with the high antioxidant activity in the seeds of these two mutants. These results show the importance of the genetic gain obtained in the mutants “US2-6” and “ML2-37”, for TPC and lignan content, which can be used as a promising source of good nutrients.

With regard to TFC, a fluctuation from 7.18 to 16.09 mg EC/g was observed, which is higher than the variations reported by Lin et al. [30] (7.14–13.54 mg EC/g) and Zhou et al. [45] (5.8–8.04 mg EC/g). However, our values remain lower than those found by Reshma et al. [52] (17.54–20.57 mg EC/g) and by Samuel and Genevieve [53] (18 mg EC/g). The white seeds of the mutant lines “US1-2” (16.09 mg EC/g) and “US2-1” (15.69 mg EC/g) showed a genetic gain of 3.7 and 3.2 mg CE/g in TFC, compared to the wild-type parent “US06” (12.41 mgEC/g). Besides this, the mutant “US1-2” also recorded a genetic gain of 0.55% in crude fibers and 1.11 mg in lignan content, compared to the parent “US06”. This demonstrates that mutagenesis breeding has brought together more than one qualitative trait in a single genotype (US1-2), which clearly benefits the variety selection process. This suggests the possibility of using the “US1-2” genotype as a relevant germopalsm to develop varieties carrying a set of desirable traits.

The free radical scavenging activity (FRSA) ranged from 35.44 to 78.62%, which is broader than the variation recorded among several Moroccan sesame cultivars collected from different cultivation areas (59.67–64.17%) [25]. The extracts from the black seeds of the mutant “US2-6” (78.62%) and the brown seeds of the mutant “ML2-37” (73.47%) showed the highest ability to stabilize DPPH radicals via the electron donor mechanism. This is in agreement with previous studies that showed that the antioxidant activity of black sesame seeds is higher than that of white ones [44,54,55,56]. Recent studies have shown that black sesame seeds can be a good source of phytonutrients that can have a positive impact on human health [57,58]. The high antioxidant capacity of these mutants may be explained by their richness in polyphenols, anthocyanins, and flavonoids, as shown by the strong and positive correlation between FRSA and TPC (r = 0.850 ***), FRSA and TAC (r = 0.712 ***), and FRSA and TFC (r = 0.656 **). This is in perfect agreement with previous studies that showed that the high amounts of phenolic compounds, anthocyanins, and flavonoids in sesame seed are responsible for its high antioxidant capacity [45,59,60,61]. However, there are reports in the literature that show that the antioxidant activities in sesame may also be related to the presence of certain peptides in its seeds [62,63]. Therefore, it would be interesting to further analyze black seeds from the mutant “US2-6” and brown seeds from the mutant “ML2-37” for their content of antioxidant peptides. Anyway, our findings suggest that seeds of both mutant lines may be relevant and valuable sources for scavenging free radicals.

Finally, the content of total anthocyanin (TAC) in sesame mutant seeds was investigated for the first time in the present study. Our results show that anthocyanin existed only in pigmented sesame seeds, recording the highest levels in the mutants “US2-6” (black seed), “ML2-68” (grey seed) and “ML2-37” (brown seed). This is in agreement with previous works reporting that anthocyanins are concentrated in the epidermal palisade coat of black soybean seeds and contribute to their pigmentation [64,65,66,67,68,69]. Compared to non-pigmented genotypes, the mutants “US2-6”, “ML2-68” and “ML2-37” were the most anthocyanin-rich, with 0.35, 0.27, and 0.27 mg/g, respectively, which suggests they are promising mutant lines with high antioxidant properties and medicinal value. In a previous work, a much lower TAC of 0.0126 mg/g was reported in raw sesame seed [70], confirming, thus, the relevance and particular interest of those three sesame mutants. However, further investigation is needed to analyze the total anthocyanin metabolites and their stability in these mutant lines.

One major goal of plant breeding and selection is to gather the maximum number of beneficial traits or characteristics in a variety. However, conventional breeding techniques do not allow for easily reaching this objective, as usually additional efforts of trait recombination through crosses are required. This is why the present work is very interesting, since mutagenesis breeding, using the EMS chemical agent, has led to the improvement of a set of biochemical traits in the same line. This is the case of the mutants “US2-6” and “ML2-37”, which showed a genetic gain in TPC, TAC, lignans and FRSA, making them rich in antioxidant substances and activity. This genetic gain is more interesting than what has been mostly reported in the international literature. In particular, when compared to the performance of the existing Moroccan cultivars [25,27], these findings highlight the relevant and valuable genetic progress achieved in some quality traits in Moroccan sesame. A few previous studies elsewhere reported similar or even better performances in some germplasms; however, only for some particular and individual traits. Furthermore, access to natural and foreign germplasms is more and more complicated due to the restricted exchange and increased competition among international research centers or institutions. Therefore, mutagenesis breeding is considered a good and efficient strategy to broaden the existing genetic variability, enhance the national genetic background and germplasm, and speed up breeding and varieties release.

## 4. Materials and Methods

### 4.1. Plant Material

The plant material used in this study consisted of 13 M_3_ sesame mutant lines, along with their wild-type parents, “US06” and “ML13”. The genotype “ML13” is a Moroccan cultivar commonly grown in Morocco, mainly in the Tadla area; it is characterized by one capsule/leaf axil, bicarpellate capsules, and indeterminate growth. The genotype “US06” is a Mexican cultivar (accession PI561704), kindly provided by USDA-ARS, Griffin, GA, USA and it is used as a germplasm for our sesame breeding program. It has three capsules/leaf axil, bicarpellate capsules, and indeterminate growth. The 13 mutants of the M_3_ generation were developed from these two parents (ML13 and US06) through mutagenesis breeding using the chemical agent ethyl methane sulfonate (EMS) [23]. These mutant lines have exhibited some valuable morphological and agronomic traits, as reported by Kouighat et al. [22]. In addition, some of these mutants are drought-tolerant during germination and early seedling growth stages, as shown in our recent study [23]. Therefore, it is relevant and interesting to assess whether these mutants also show a genetic gain, compared to the parents, in terms of certain quality and nutritional traits. The main seed characteristics of these plant materials are summarized in the Table 3. Seeds of the M_2_ mutants, previously selected, were planted in pots at the Regional Center of Agricultural Research in Meknes, INRA-Morocco (33°83′ N, 5°42′ W), for M_3_ seed production and evaluation. The sowing was done on 4 May 2020, the derived plants were grown under the same environmental conditions, and the harvest was done on 5-9 October 2020 when the plants reached their maturity. Each genotype was represented by three plants from which M_3_ seeds were randomly sampled for processing and analysis. After being harvested, the parents and M_3_ seeds were conserved under room temperature and humidity conditions for a short time, before being used in this study. At the time of their processing, seed samples showed an average moisture content of about 9.2%, with a variation from 8 to 11%, as indicated in the Table 3.

### 4.2. Chemical and Biochemical Seeds Analysis

For all the parameters studied, dry, healthy, and uniform seeds were analyzed in three replicates.

#### 4.2.1. Ash Content

Ash content (%) was determined according to the protocol of Chemists and Horwitz [71]. For each sample, 5 g of seed was weighed in a crucible, placed in an oven and incinerated at 800 °C for 6 h. The weight of the residue was expressed as ash content.

#### 4.2.2. Carbohydrate Content

Seed samples were analyzed for carbohydrate content (%) following the standard procedure of Chemists and Horwitz [71].

#### 4.2.3. Protein Content

To determine the crude protein content (%), a quantity of 0.2 g from each sample was ground and then weighed. Crude protein percentage was determined by the Kjeldahl method according to the protocol of Energy [72].

#### 4.2.4. Crude Fiber Content

Crude fiber content (%) was estimated by the AOAC [73] Standard Protocol. Briefly, 3 g of sample underwent segmental thermal digestion with dilute acid and alkaline solutions.

For the rest of the parameters analyzed, seed extracts of the experimented 15 sesame lines were prepared according to the protocol of Vishwanath et al. [74], with some modifications—10 g of seeds of each line were ground separately and suspended in 100 mL of ethanol (90%) and kept for 2 h under agitation. Afterward, the samples were filtered and subjected to vacuum evaporation, and re-dissolved in 2 mL ethanol (90%).

#### 4.2.5. Total Phenol Content

Total polyphenol content (TPC) was determined by the method of Kao and Chen [75], with some modifications. Briefly, 0.5 g of each seed extract was dissolved in 5.0 mL of methanol and added to 0.5 mL of Folin–Ciocalteau reagent for 5 min. Before incubation for 1 h at room temperature, 1 mL of sodium carbonate (1N) was added. The absorbance was measured at 750 nm by a spectrophotometer. The calibration curve was prepared using 100, 200, 400, 600, 800 and 1000 µg/mL solutions of gallic acid (standard phenolic compound) in methanol. The total phenol contents were presented as milligrams of gallic acid per gram (mg GAE/g) of the sample dry weight.

#### 4.2.6. Total Flavonoid Content

Total flavonoid content (TFC) was determined by aluminum chloride colorimetric assay as described by Dravie et al. [61]. In test tubes, 60 µL aliquots of each extract were added to 2 mL of ethanol. A quantity of 150 µL of 5% sodium nitrite (NaNO_2_) solution was added to each mixture and allowed to stand for 5 min before the addition of 150 µL of 10% aluminum chloride (AlCl_3_). The resulting mixture was allowed to stand for an additional 5 min and then 1 mL of 1 M sodium hydroxide (NaOH) was added and vortexed for 10 s. The absorbance was measured at 510 nm against the prepared reagent blank and the total flavonoid content was expressed as catechin equivalent (mg CE/g) on dry matter.

#### 4.2.7. Total Anthocyanin Content

Total anthocyanin content (TAC) was measured according to the protocol of Ryu and Koh [76] using the differential pH method. Sesame extracts were diluted with a buffer solution of 25 mM potassium chloride (pH 1.0) and 0.4 M sodium acetate (pH 4.5). The absorbance was measured by an ultraviolet–visible (UV-Vis) spectrophotometer at 520 nm and 700 nm. In each extract, the total anthocyanins content was expressed as mg cyanidin-3-O-glucoside equivalent per gram (mg/g) in sesame seeds.

Total anthocyanin content = A × MW × DF × 10^3^/ε × 1

where A = (A_520nm_ − A_700nm_) pH 1.0 − (A_520nm_ − A_700nm_) pH 4.5; MW is the molecular weight of cyanidin-3-glucoside (449.2 g/mol), DF is the dilution factor and ε is the molar extinction coefficient of cyanidin-3-glucoside (26,900 L/mol × cm).

#### 4.2.8. Total Lignan Content

The total lignan content (mg/g) was measured by UV-Vis spectrophotometer at 290 nm in the seed extracts according to the procedure of Kim et al. [77].

#### 4.2.9. Free Radical Scavenging Activity

To measure the antioxidant activity (FRSA) of sesame extracts, the DPPH (diphenyl-2-picryl hydrazyl) method was used as described by Dravie et al. [61]. The experiment was repeated three times and the mean values were reported. The control containing all solutions except the extract was also maintained. The absorbance was done at 517 nm and the radical scavenging activity was calculated and expressed as a percentage of the control using the following formula:

% Scavenging [DPPH] = [(A_0_ − A_1_)/A_0_] × 100

where A_0_ is the absorbance of the blank (in which the same volume of methanol was used instead of the sample) and A_1_ was the absorbance of sesame seed extract.

### 4.3. Statistical Analysis

The gathered data were subjected to analysis of variance (ANOVA) to test the significance of differences among the genotypes studied (mutant lines and parents). In the event that significant differences were observed for certain parameters, Duncan’s new multiple range test (DMRT) was performed to compare and group the mutant lines. Besides, planned contrasts (one-way ANOVA) were used to test the significance of differences observed between the wild-type parents (ML13 and US06) and their respective mutants (The parent ML13 vs. ML-mutants and the parent US06 vs. US-mutants), and between the both ML and US types (ML-genotypes vs. US-genotypes). Finally, a correlation analysis among the analyzed parameters was done using Pearson’s coefficient [45]. All the analyses were carried out using the statistical software package SPSS (version 20).

## 5. Conclusions

The present work targeted some chemical and biochemical attributes to assess the genetic progress achieved in 13 sesame mutant lines (M_3_ generation), compared to the existing cultivar (ML13) and germplasm (US06) in Morocco. Among these attributes, the content of anthocyanins in sesame mutant seeds has been approached so far for the first time. Our findings have revealed a high genetic variation among the mutant lines studied for most of the investigated traits, suggesting the effectiveness of mutagenesis breeding for improving seed quality and nutritional attributes in sesame. More interestingly, some particular mutants have shown a genetic gain for more than one trait, which is beneficial for sesame breeding as a medicinal and oilseed plant. These quirky mutant lines will be used as valuable germplasms for selecting and releasing new cultivars with nutritional quality and potential medicinal properties.

## Figures and Tables

**Table 1:** Results of analysis of variance for 15 sesame genotypes (13 mutant lines and their 2 parents) evaluated for various quality traits (mean square, value of contrast and level of difference significance).

Source of Variation	DF	Sugar	Protein	Ash	Fiber	TFC	TPC	TAC	Lignans	FRSA
Mean square										
Genotype	14	56.504 **	36.186 **	0.290	1.044 **	35.749 ***	1.753 ***	0.143 **	1.762 ***	440.997 ***
Contrast value										
ML13_parent_ vs. ML_mutants_	1	3.91 **	2.25 **	-	0.14 **	9.08 ***	0.93 **	2.15 ***	2.3 *	66.38 ***
US06_parent_ vs. US_mutants_	1	64.58 ***	1.91 **	-	7.83 ***	34.01 ***	9.76 ***	4.05 ***	7.31 ***	95.25 ***
US_genotypes_ vs. ML_genotypes_	1	21.52 ***	14.50 **	-	3.21 ***	3.86 ***	11.46 ***	1.89 ***	9.13 ***	52.46 ***

DF is the degree of freedom. *, ** and ***: significant differences at 5%, 1%, and 1‰ probability levels, respectively.

**Table 2:** Correlation coefficients among the biochemical traits studied in 13 M_3_ sesame mutants and their wild-type parents.

	TFC	TPC	TAC	Lignans	FRSA
TFC	1	0.398 **	0.278	0.254	0.656 **
TPC		1	0.479 **	0.398 **	0.850 ***
TAC			1	0.303 *	0.712 ***
Lignans				1	0.312 *
FRSA					1

TFC, total flavonoids content; TPC, total polyphenols content; TAC, total anthocyanins content; FRSA, free antiradical scavenging activity. *, **, *** significant differences at 5%, 1% and 1‰ probability level, respectively.

**Table 3:** Main phenotypic characteristics of the sesame genotypes studied and seeds’ moisture content during processing.

Lines	Characteristics	Seed Moisture Content (%)
“US06”	Parent (wild-type), white seeds	9.2
“US2-7”	Mutant, white seeds	9.3
“US1-DL”	Mutant, white seeds	9.7
“US1-3”	Mutant, white seeds	9.0
“US2-1”	Mutant, white seeds	11.0
“US1-2”	Mutant, white seeds	9.4
“US2-6”	Mutant, black seeds	9.1
“US2-6-1”	Mutant, white seeds	8.7
“US2-6-2”	Mutant, white seeds	10.2
“ML13”	Parent (wild-type), beige seeds	8.7
“ML2-5”	Mutant, brown seeds, tolerant to severe drought during germination	8.0
“ML2-10”	Mutant, brown seeds, tolerant to severe drought during germination	8.9
“ML2-72”	Mutant, brown seeds	9.5
“ML2-37”	Mutant, brown seeds, tolerant to severe drought during germination	8.7
“ML2-68”	Mutant, grey seeds	9.1

## Data Availability

Not applicable.

## References

[1] Pal D., Chandra P., Sachan N. (2020). Sesame seed in controlling human health and nutrition. Nuts and Seeds in Health and Disease Prevention.

[2] Sani I., Okpalaoka C.C., Bello F., Warra A.A., Abdulhamid A. (2014). Flavonoid content and antioxidant potential of white and brown sesame seed oils. Eur. J. Biomed. Pharm..

[3] Shah N.C. (2016). Sesamum indicum (Sesame or Til): Seeds and Oil-A historical and scientific evaluation from Indian perspective. Asian Agric.-Hist..

[4] Dossa K., Li D., Wang L., Zheng X., Yu J., Wei X., Fonceka D., Diouf D., Liao B., Cisse N. (2017). Dynamic transcriptome landscape of sesame (*Sesamum indicum* L.) under progressive drought and after rewatering. Genom. Data.

[5] Zhang Y., Wang L., Gao Y., Li D., Yu J., Zhou R., Zhang X. (2018). Genetic dissection and fine mapping of a novel Dt gene associated with determinate growth habit in sesame. BMC Genet..

[6] Nahar L., Xiao J., Sarker S.D. (2019). Introduction of phytonutrients. Handbook of Dietary Phytochemicals.

[7] Connor A.M., Luby J.J., Tong C.B., Finn C.E., Hancock J.F. (2002). Genotypic and environmental variation in antioxidant activity, total phenolic content, and anthocyanin content among blueberry cultivars. J. Am. Soc. Hortic. Sci..

[8] Lampart-Szczapa E., Korczak J., Nogala-Kalucka M., Zawirska-Wojtasiak R. (2003). Antioxidant properties of lupin seed products. Food Chem..

[9] Dar A.A., Arumugam N. (2013). Lignans of sesame: Purification methods, biological activities and biosynthesis—A review. Bioorg. Chem..

[10] Peterson J., Dwyer J. (1998). Flavonoids: Dietary occurrence and biochemical activity. Nutr. Res..

[11] Sarker S.D., Nahar L. (2007). Chemistry for Pharmacy Students.

[12] Yao L.H., Jiang Y.-M., Shi J., Tomas-Barberan F.A., Datta N., Singanusong R., Chen S.S. (2004). Flavonoids in food and their health benefits. Plant Foods Hum. Nutr..

[13] Abaza L., Msallem M., Daoud D., Zarrouk M. (2002). Caractérisation des huiles de sept varietes d’olivier tunisiennes. Oléagineux Corps Gras Lipides.

[14] Fawole O.A., Amoo S.O., Ndhlala A.R., Light M.E., Finnie J.F., Van Staden J. (2010). Anti-inflammatory, anticholinesterase, antioxidant and phytochemical properties of medicinal plants used for pain-related ailments in South Africa. J. Ethnopharmacol..

[15] Zhang H., Miao H., Wei L., Li C., Zhao R., Wang C. (2013). Genetic analysis and QTL mapping of seed coat color in sesame (*Sesamum indicum* L.). PLoS ONE.

[16] Kong A.-N.T., Yu R., Chen C., Mandlekar S., Primiano T. (2000). Signal transduction events elicited by natural products: Role of MAPK and caspase pathways in homeostatic response and induction of apoptosis. Arch. Pharm. Res..

[17] Wang D., Zhang L., Huang X., Wang X., Yang R., Mao J., Wang X., Wang X., Zhang Q., Li P. (2018). Identification of Nutritional Components in Black Sesame Determined by Widely Targeted Metabolomics and Traditional Chinese Medicines. Molecules.

[18] Arslan Ç., Uzun B., Ülger S., İlhan Çağırgan M. (2007). Determination of oil content and fatty acid composition of sesame mutants suited for intensive management conditions. J. Am. Oil Chem. Soc..

[19] Madhusudan K., Nadaf H.L., Motagi B.N., Singh S. (2008). Induced mutants with improved nutraceutical traits in sesame (*Sesamum indicum* L.). Proceedings of the International Symposium on Induced Mutations in Plants (ISIM).

[20] Masur S., Madhusudan K. (2009). Characterization of induced mutants of sesame (Sesamum indicum L.) for confectionary and quality traits. Proceedings of the International Conference on Peaceful Uses of Atomic Energy-2009.V.2.

[21] El Harfi M., Jbilou M., Hanine H., Rizki H., Fechtali M., Nabloussi A. (2018). Genetic diversity assessment of moroccan sesame (*Sesamum indicum* L.) populations using agro-morphological traits. J. Agric. Sci. Technol. A.

[22] El Harfi M., Charafi J., Houmanat K., Hanine H., Nabloussi A. (2021). Assessment of genetic diversity in moroccan sesame (*Sesamum indicum*) using ISSR molecular markers. OCL.

[23] Kouighat M., Channaoui S., Labhilili M., El Fechtali M., Nabloussi A. (2020). Novel genetic variability in sesame induced via ethyl methane sulfonate. J. Crop Improv..

[24] Kouighat M., Hanine H., El Fechtali M., Nabloussi A. (2021). First report of sesame mutants tolerant to severe drought stress during germination and early seedling growth stages. Plants.

[25] Rizki H., Kzaiber F., Elharfi M., Nablousi A., Hanine H. (2014). Chemical composition and morphological markers of 35 cultivars of sesame (*Sesamum indicum*. L) from different areas in Morocco. Int. J. Sci. Res..

[26] Gharby S., Harhar H., Bouzoubaa Z., Asdadi A., El Yadini A., Charrouf Z. (2017). Chemical characterization and oxidative stability of seeds and oil of sesame grown in Morocco. J. Saudi Soc. Agric. Sci..

[27] Nabloussi A., Hanine H., Harfi M.E., Rizki H., Méndez-Vilas A. (2017). Moroccan sesame: An overview of seed and oil quality. Science within Food: Up-to-Date Advances on Research and Educational Ideas.

[28] Yousuf B., Gul K., Wani A.A., Singh P. (2016). Health benefits of anthocyanins and their encapsulation for potential use in food systems: A review. Crit. Rev. Food Sci. Nutr..

[29] Khoo H.E., Azlan A., Tang S.T., Lim S.M. (2017). Anthocyanidins and anthocyanins: Colored pigments as food, pharmaceutical ingredients, and the potential health benefits. Food Nutr. Res..

[30] Lin X., Zhou L., Li T., Brennan C., Fu X., Liu R.H. (2017). Phenolic content, antioxidant and antiproliferative activities of six varieties of white sesame seeds (*Sesamum indicum* L.). RSC Adv..

[31] El Khier M.K.S., Ishag K.E.A., Yagoub A.A. (2008). Chemical composition and oil characteristics of sesame seed cultivars grown in Sudan. Res. J. Agric. Biol. Sci..

[32] Yaseen G., Ahmad M., Zafar M., Akram A., Sultana S., Ahmed S.N., Kilic O. (2021). Sesame (*Sesamum indicum* L.). Green Sustainable Process for Chemical and Environmental Engineering and Science.

[33] Zebib H., Bultosa G., Abera S. (2015). Physico-chemical properties of sesame (*Sesamum indicum* L.) varieties grown in Northern Area, Ethiopia. Agric. Sci..

[34] Gandhi A.P., Srivastava J. (2007). Studies on the production of protein isolates from defatted sesame seed (*Sesamum indicum*) flour and their nutritional profile. ASEAN Food J..

[35] Gadade B.V., Kachare D.P., Satbhai R.D., Naik R.M. (2017). Nutritional composition and oil quality parameters of sesame (*Sesamum indicum* L.) genotypes. Int. Res. J. Multidiscip. Stud..

[36] Hassan M.A. (2012). Studies on Egyptian sesame seeds (*Sesamum indicum* L.) and its products 1-physicochemical analysis and phenolic acids of roasted Egyptian sesame seeds (*Sesamum indicum* L.). World J. Dairy Food Sci..

[37] Moazzami A., Kamal-Eldin A. (2009). Sesame seed oil. Gourmet and Health-Promoting Specialty Oils.

[38] Ünal M.K., Yalçın H. (2008). Proximate composition of Turkish sesame seeds and characterization of their oils. Grasas Aceites.

[39] Nzikou J.M., Matos L., Bouanga-Kalou G., Ndangui C.B., Pambou-Tobi N.P.G., Kimbonguila A., Silou T., Linder M., Desobry S. (2009). Chemical composition on the seeds and oil of sesame (*Sesamum indicum* L.) grown in Congo-Brazzaville. Adv. J. Food Sci. Technol..

[40] Ogbonna P.E., Ukaan S.I. (2013). Chemical composition and oil quality of seeds of sesame accessions grown in the Nsukka Plains of South Eastern Nigeria. Afr. J. Agric. Res..

[41] Obiajunwa E.I., Adebiyi F.M., Omode P.E. (2005). Determination of essential minerals and trace elements in Nigerian sesame seeds, using TXRF technique. Pak. J. Nutr..

[42] Abbas S., Sharif M.K., Butt M.S., Shahid M. (2020). Screening of Pakistani sesame cultivars for nutritive value and bioactive components. Pak. J. Agric. Sci..

[43] Khan I.U., Rathore B.S., Syed Z. (2019). Evaluation of polyphenols, flavonoids and antioxidant activity in different solvent extracts of sesame (*Sesamum indicum* L.) genotypes. Int. J Seed Spices.

[44] Kurt C., Uçar B., Akkaya M.R. (2020). Determination of total phenolic content and antioxidant activities of different sesame (*Sesamum indicum* L.) genotypes. Turk. J. Agric.-Food Sci. Technol..

[45] Zhou L., Lin X., Abbasi A.M., Zheng B. (2016). Phytochemical contents and antioxidant and antiproliferative activities of selected black and white sesame seeds. BioMed Res. Int..

[46] Ha T.J., Lee M.-H., Seo W.D., Baek I.-Y., Kang J.E., Lee J.H. (2017). Changes occurring in nutritional components (phytochemicals and free amino acid) of raw and sprouted seeds of white and black sesame (*Sesamum indicum* L.) and screening of their antioxidant activities. Food Sci. Biotechnol..

[47] Shahidi F., Liyana-Pathirana C.M., Wall D.S. (2006). Antioxidant activity of white and black sesame seeds and their hull fractions. Food Chem..

[48] Singh K.K., Kanbi V.H., Chaudhary M.K. (2015). Nutritional evaluation of different varieties of sesame (*Sesamum indicum* L.). Indian J. Nutr. Diet..

[49] Yoshioka Y., Li X., Zhang T., Mitani T., Yasuda M., Nanba F., Toda T., Yamashita Y., Ashida H. (2017). Black soybean seed coat polyphenols prevent AAPH-induced oxidative DNA-damage in HepG2 cells. J. Clin. Biochem. Nutr..

[50] Moazzami A.A., Haese S.L., Kamal-Eldin A. (2007). Lignan contents in sesame seeds and products. Eur. J. Lipid Sci. Technol..

[51] Shi L.-K., Liu R.-J., Jin Q.-Z., Wang X.-G. (2017). The contents of lignans in sesame seeds and commercial sesame oils of China. J. Am. Oil Chem. Soc..

[52] Reshma M.V., Namitha L.K., Sundaresan A., Ravi Kiran C. (2013). Total phenol content, antioxidant activities and α-glucosidase inhibition of sesame cake extracts. J. Food Biochem..

[53] Samuel N.C., Genevieve A.C. (2017). Proximate analysis and phytochemical properties of sesame (*Sesamum indicum* L.) seeds grown and consumed in Abakaliki, Ebonyi State, Nigeria. Int. J. Health Med..

[54] Kuo P.-C., Lin M.-C., Chen G.-F., Yiu T.-J., Tzen J.T. (2011). Identification of methanol-soluble compounds in sesame and evaluation of antioxidant potential of its lignans. J. Agric. Food Chem..

[55] Botelho J.R.S., Medeiros N.G., Rodrigues A.M., Araujo M.E., Machado N.T., Santos A.G., Santos I.R., Gomes-Leal W., Junior R.N.C. (2014). Black sesame (*Sesamum indicum* L.) seeds extracts by CO_2_ supercritical fluid extraction: Isotherms of global yield, kinetics data, total fatty acids, phytosterols and neuroprotective effects. J. Supercrit. Fluids.

[56] Das R., Bhattacharjee C. (2015). Processing sesame seeds and bioactive fractions. Processing and Impact on Active Components in Food.

[57] Sun S., Zhang X., Chen W., Zhang L., Zhu H. (2016). Production of natural edible melanin by auricularia auricula and its physicochemical properties. Food Chem..

[58] Saisum S., Hudthagosol C., Srisorrachatr S. (2020). Effect of black sesame seeds (*Sesamum indicum* L.) consumption on sleep quality among Thai elderly. Food Appl. Biosci. J..

[59] Lee K.J., Lee J.-R., Ma K.-H., Cho Y.-H., Lee G.-A., Chung J.-W. (2016). Anthocyanin and isoflavone contents in Korean black soybean landraces and their antioxidant activities. Plant Breed. Biotechnol..

[60] Hussain S.A., Hameed A., Ajmal I., Nosheen S., Suleria H.A.R., Song Y. (2018). Effects of sesame seed extract as a natural antioxidant on the oxidative stability of sunflower oil. J. Food Sci. Technol..

[61] Dravie E.E., Kortei N.K., Essuman E.K., Tettey C.O., Boakye A.A., Hunkpe G. (2020). Antioxidant, phytochemical and physicochemical properties of sesame seed (*Sesamum indicum* L.). Sci. Afr..

[62] Shao J., Zhang G., Fu J., Zhang B. (2020). Advancement of the preparation methods and biological activity of peptides from sesame oil byproducts: A review. Int. J. Food Prop..

[63] Lu X., Song G., Huang J., Zhang L., Sun Q. (2019). Extraction, identification and structure-activity relationship of antioxidant peptides from sesame (*Sesamum indicum* L.) protein hydrolysate. Food Res. Int..

[64] Dixit A.K., Bhatnagar D., Kumar V., Rani A., Manjaya J.G., Bhatnagar D. (2010). Gamma irradiation induced enhancement in isoflavones, total phenol, anthocyanin and antioxidant properties of varying seed coat colored soybean. J. Agric. Food Chem..

[65] Kristamtini K., Wiranti E.W. (2017). Clustering of 18 local black rice base on total anthocyanin. Biol. Med. Nat. Prod. Chem..

[66] Malenčić D., Cvejić J., Miladinović J. (2012). Polyphenol content and antioxidant properties of colored soybean seeds from central Europe. J. Med. Food.

[67] Todd J.J., Vodkin L.O. (1993). Pigmented soybean (*Glycine max*) seed coats accumulate proanthocyanidins during development. Plant Physiol..

[68] Xu B., Chang S.K. (2008). Antioxidant capacity of seed coat, dehulled bean, and whole black soybeans in relation to their distributions of total phenolics, phenolic acids, anthocyanins, and isoflavones. J. Agric. Food Chem..

[69] Zhang Y., Sun J., Zhang X., Wang L., Che Z. (2011). Analysis on genetic diversity and genetic basis of the main sesame cultivars released in China. Agric. Sci. China.

[70] Rababah T.M., Al-U’datt M., Al-Mahasneh M., Obaidat M., Almajwal A., Odeh A., Brewer S., Yang W. (2016). Effect of tehina processing and storage in the physical-chemical quality. Int. J. Agric. Biol. Eng..

[71] Chemists A., Horwitz W. (1975). Official Methods of Analysis.

[72] Energy F.F. (2003). Methods of analysis and conversion factors. Report of a technical workshop. Food Nutr..

[73] AOAC (1994). Official Methods of Analysis.

[74] Vishwanath H.S., Anilakumar K.R., Harsha S.N., Khanum F., Bawa A.S. (2012). In vitro antioxidant activity of *Sesamum indicum* seeds. Asian J. Pharm. Clin. Res..

[75] Kao T.-H., Chen B.-H. (2006). Functional components in soybean cake and their effects on antioxidant activity. J. Agric. Food Chem..

[76] Ryu D., Koh E. (2018). Application of response surface methodology to acidified water extraction of black soybeans for improving anthocyanin content, total phenols content and antioxidant activity. Food Chem..

[77] Kim K.-S., Lee J.-R., Lee J.-S. (2006). Determination of sesamin and sesamolin in sesame (*Sesamum indicum* L.) seeds using UV spectrophotometer and HPLC. Korean J. Crop Sci..

